# Cracking Performance of Fiber-Reinforced High-RAP Asphalt Mixtures Using IDEAL-CT

**DOI:** 10.3390/ma19142936

**Published:** 2026-07-08

**Authors:** Aaditya Ojha, Hani Alzraiee, Ashraf Rahim, Shadi Saadeh, Chase Plager, Mohammad Doroudgar

**Affiliations:** 1 Civil Engineering and Construction Engineering Management Department, California State University Long Beach, Long Beach, CA 90840, USA; aaditya.ojha01@student.csulb.edu (A.O.); shadi.saadeh@csulb.edu (S.S.); shayan.doroudgar01@student.csulb.edu (M.D.); 2 Civil and Environmental Engineering Department, California Polytechnic State University, San Luis Obispo, CA 93407, USA; arahim@calpoly.edu (A.R.); cplager@calpoly.edu (C.P.)

**Keywords:** Hot-Mix Asphalt (HMA), Reclaimed Asphalt Pavement (RAP), Polymer Fiber (PF), Cracking Tolerance Index (CTI)

## Abstract

**Highlights:**

- Fibers improved IDEAL-CT cracking resistance in 15%, 25%, and 40% RAP mixes.
- The best results depended on RAP level, fiber type, and dosage.
- 25% RAP showed the largest significant CTIndex gains with fiber addition.
- Fiber dosage should be optimized for each RAP content, not applied uniformly.
- Commercial para-aramid fibers can improve lab cracking resistance of high-RAP HMA.
- Field validation and added mechanistic tests are needed before broad implemen-tation.

**Abstract:**

High reclaimed asphalt pavement (RAP) mixtures can improve pavement sustainability by reducing virgin binder and aggregate demand, but high RAP contents may increase mixture stiffness and reduce cracking tolerance. This study evaluates whether commercially available para-aramid fibers can improve the intermediate-temperature cracking resistance of high-RAP hot-mix asphalt using the IDEAL-CT test. Two para-aramid fiber products, a wax-coated fiber and an emulsion-treated fiber, were evaluated at dosages of 0.05%, 0.10%, and 0.15% by total mixture weight in asphalt mixtures containing 15%, 25%, and 40% RAP. The results showed that fiber effectiveness depended strongly on RAP content, fiber treatment, and dosage. The 25% RAP mixture had the lowest control CTIndex and showed the greatest improvement from fiber addition. In this group, 0.10% wax-coated fiber increased CTIndex by 170%, while 0.15% emulsion-treated fiber increased CTIndex by 263%. For the 15% RAP mixture, 0.05% emulsion-treated fiber and 0.10% wax-coated fiber produced statistically significant improvements. For the 40% RAP mixture, 0.10% emulsion-treated fiber produced the highest mean CTIndex among all mixtures tested, but the improvement was not statistically significant because of high specimen variability. Overall, the findings indicate that para-aramid fibers can improve laboratory cracking resistance in RAP mixtures, but the optimum dosage is mixture-specific and should not be applied uniformly across RAP contents. Because this study was limited to Ideal-CT, additional rutting, fatigue, aging, workability analysis and field validation are recommended before broad implementation.

## 1. Introduction

Reclaimed asphalt pavement (RAP) is widely used in asphalt mixtures because it reduces the demand for virgin aggregate and asphalt binder while supporting more sustainable pavement construction practices [1,2]. RAP use has continued to increase in the United States, with approximately 94.6 million tons incorporated into asphalt mixtures in 2021, representing a 69% increase since 2009 [3]. In California, Caltrans has progressively allowed higher RAP contents, including 15% RAP in 2009, up to 25% RAP without blending charts by 2013, and evaluation of specifications for up to 40% RAP by 2019 [4]. However, despite these specification changes, average RAP use in California has remained relatively moderate, generally between 15% and 18% in recent years [3]. This gap between allowable RAP content and typical field use reflects continuing concerns about mixture variability and cracking performance at higher RAP contents.

The primary performance concern with high-RAP asphalt mixtures is the contribution of aged, oxidized binder from the reclaimed material. As RAP content increases, the mixture may become stiffer and less ductile, which can reduce strain tolerance and increase susceptibility to intermediate-temperature cracking if the mixture is not properly designed [1,5,6]. This concern is not only laboratory-based: FHWA has reported that state DOTs have observed premature cracking in newly constructed pavements linked to high recy-cled-binder content, reinforcing the need for performance-based evaluation of high-RAP mixtures [7]. RAP variability can also affect mixture uniformity because reclaimed materials may come from different pavement layers, maintenance histories, binder grades, and aggregate sources [5]. Therefore, although RAP provides clear sustainability and economic benefits, higher RAP contents often require performance-based evaluation and modification strategies to ensure adequate cracking resistance.

Several approaches have been used to address the cracking susceptibility of high-RAP mixtures. Recycling agents and rejuvenators are commonly used to improve the rheological properties of aged binders by restoring flexibility and improving the balance of binder fractions [8,9,10,11]. In contrast, structural modifiers such as synthetic fibers are intended to improve mixture-level cracking resistance by physically reinforcing the asphalt matrix. Previous studies have shown that fiber reinforcement can affect several mixture performance properties rather than only strength. Riccardi et al. [12] evaluated polyacrylonitrile fiber-reinforced mixtures with high RAP using rutting, stiffness, fatigue, low-temperature cracking, and SCB fracture tests; they reported improvements in rutting resistance, fatigue response, and crack-propagation resistance, suggesting that fibers act mainly by reinforcing the mixture and improving post-crack behavior.

Slebi-Acevedo et al. [13] evaluated air voids, Marshall stability, indirect tensile strength, moisture sensitivity, compactability, rutting, stiffness, and fatigue, and found that polyacrylonitrile fibers improved permanent deformation resistance, stiffness, and tensile response while maintaining comparable fatigue performance. Ramesh et al. [14] investigated the fracture response of warm-mix asphalt containing RAP and nano-glass fibers, further showing that fiber-modified RAP mixtures should be evaluated based on fracture behavior and mixture-specific dosage effects. These studies indicate that fiber benefits are linked to reinforcement, crack-bridging, energy absorption, and delayed crack propagation, but the effectiveness depends on fiber type, dosage, dispersion, and RAP content.

Laboratory cracking tests are important for evaluating these mixture-specific effects. The Indirect Tensile Asphalt Cracking Test, commonly known as IDEAL-CT, has been recommended as a practical test for assessing intermediate-temperature cracking resistance because it is relatively simple, rapid, and compatible with balanced mix design approaches [15]. IDEAL-CT provides the Cracking Tolerance Index (CTIndex), which is calculated from the load–displacement response of the specimen. Unlike peak load alone, CTIndex reflects the combined influence of fracture energy, post-peak deformation capacity, and the rate of post-peak load reduction. Therefore, IDEAL-CT can help identify whether fibers improve cracking resistance by increasing energy absorption, delaying crack opening, or reducing the brittleness of post-peak failure.

The objective of this study is to evaluate the effect of two commercially available para-aramid fiber products and three fiber dosages on the IDEAL-CT cracking response of hot-mix asphalt containing 15%, 25%, and 40% RAP. The study specifically compares wax-coated and emulsion-treated para-aramid fibers at dosages of 0.05%, 0.10%, and 0.15% by total mixture weight. The contribution of this work is the evaluation of the interaction between RAP content, fiber type, and fiber dosage using CTIndex and load–displacement behavior. This approach provides practical guidance for optimizing fiber dosage in high-RAP mixtures and identifies the need for additional mechanistic, durability, and field validation before broad implementation.

## 2. Materials and Methods

### 2.1. Materials

This study investigated three distinct HMA mixtures, each incorporating a different percentage of RAP. The first mixture contained 15% RAP and was supplied by CalPortland (Las Vegas, NV, USA), utilizing a PG 64-10 asphalt binder. This particular mix is typically employed in projects located along California’s Central Coast, with the job mix formula (JMF) indicating an optimum binder content of 5.6% by mix total weight. The second and third mixtures contained 25% and 40% RAP, respectively, both supplied by a different supplier, i.e., Granite Construction (Watsonville, CA, USA), and utilized a PG 58-22 asphalt binder. These mixtures are commonly used in pavement projects in the Bay Area. As per the provided JMFs, the 25% RAP mix had an optimum binder content of 4.8%, whereas the 40% RAP mix was formulated with a slightly higher optimum binder content of 5.2%.

For all three mixtures, the respective construction companies directly provided the JMFs, including aggregate gradations and binder contents. Additionally, each mixture included a liquid antistripping agent, introduced at a consistent dosage rate of 0.5% by weight of the asphalt binder, to enhance resistance to moisture damage. Detailed aggregate gradations employed in these mixes are summarized and presented in Table 1.

The volumetric properties of the three job mix formulas (JMFs) are as follows. The 15% RAP mix had an Optimum Asphalt Content (OAC) of 5.6%, Voids in the Mineral Aggregate (VMA) of 14.5%, Voids Filled with Asphalt (VFA) of 71.7%, and design air voids (Va) of 4.1%. The 25% RAP mix had an OAC of 4.8%, VMA of 13.9%, VFA of 67.6%, and Va of 4.5%. The 40% RAP mix had an OAC of 5.2%, VMA of 14.7%, VFA of 72.8%, and Va of 4.0%. All three mixes satisfy Superpave volumetric design requirements for their respective traffic levels and binder grades.

The VMA values are comparable across the three mixes, ranging narrowly from 13.9% to 14.7%, indicating that the differences in OAC (4.8% to 5.6%) are driven by the varying aged binder contribution from RAP rather than by differences in aggregate skeleton packing or particle interlocking geometry. This consistency in VMA confirms that the aggregate structures of the three mixes are fundamentally similar, and that observed differences in Cracking Tolerance Index (CTIndex) values across RAP levels can be attributed to binder stiffness and fiber interaction effects rather than volumetric design variations. Additionally, specimen air voids were controlled to 7.0% ± 0.5% during compaction for all mix conditions, further minimizing the influence of void structure on cracking performance results.

**Figure 1:**
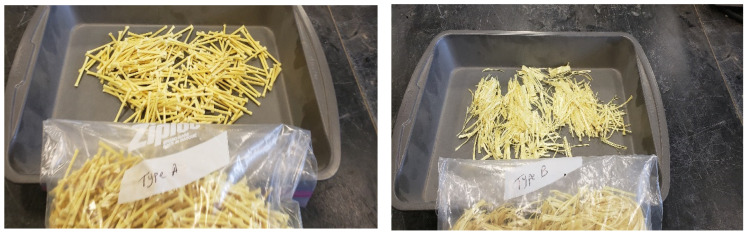
Fibers used for the study: Fiber Type A (**left**) and Type B (**right**) (Rahim et al. 2025 [6]).

Two types of polymer fibers, previously used in a Caltrans maintenance project on State Route 1, were investigated. As shown in Figure 1, two commercial para-aramid fiber products were evaluated: (i) wax-coated para-aramid fiber (37% coating by weight) and (ii) para-aramid fiber with emulsion treatment (25% treatment by weight). These fibers were introduced into the HMA mixtures at three different dosages: 0.05%, 0.10%, and 0.15% of the total HMA weight. Control mixtures, without any fiber addition, were also prepared for each RAP content to serve as a baseline. It is noted that the 40% RAP mixture was only tested for Fiber Type B.

Both fiber products were commercial para-aramid fibers with different surface treatments. Fiber Type A contained a minimum of 63% para-aramid by weight with a wax coating of up to 37%, while Fiber Type B contained a minimum of 75% para-aramid by weight with a liquid emulsion binder treatment of up to 25%. Both fibers were yellow, had a nominal length of either 19 mm or 38 mm with a ±10% tolerance, tensile strength greater than 2700 MPa, Young’s modulus greater than 80 GPa, and decomposition temperature above 425 °C. These properties indicate that both fibers have sufficient thermal stability for asphalt mixing and high tensile stiffness for potential crack-bridging reinforcement.

The naming for the samples in this study includes the RAP content, fiber type, and fiber dosage. For example, “15-A0.10” indicates a mix with 15% RAP, Fiber A, and 0.10% fiber content. Control samples are labeled with a “C”, such as “25-C”, representing a mix with 25% RAP and no fiber.

### 2.2. Specimen Preparation

Specimens for the Cracking Tolerance Index (CTI) tests were prepared using a Superpave Gyratory Compactor in accordance with AASHTO T 312 [16]. For each mixture, the required virgin aggregates and RAP materials were first dried to constant mass, weighed separately in steel pans according to the corresponding job mix formula, and then combined to obtain the target batch weight. For the specimens, approximately 5250 g of aggregate blend was typically prepared to produce two compacted specimens. The batched aggregates, including RAP, and the asphalt binder were heated in an oven to the appropriate mixing temperature based on the binder grade. Once the materials reached the target temperature, the heated aggregate blend was placed in a mechanical mixer, and a crater was formed at the center of the aggregate mass to allow uniform binder distribution. The required liquid antistripping agent, used at 0.5% by weight of asphalt binder, was added to the binder before mixing.

The asphalt binder and the selected fiber dosage were then introduced into the aggregate blend and mixing continued until all aggregate particles were uniformly coated and the fibers were evenly dispersed throughout the mixture. After mixing and short-term conditioning, the amount of mixture required for compaction was calculated using the theoretical maximum specific gravity, target specimen volume, and target air void content of 7.0 ± 0.5% using an iterative process for CTI testing. Trial specimens were prepared at the calculated mass, as well as at W + 10 g and W − 10 g, to refine the exact mass needed to achieve the required air void range. Final specimens were compacted to 150 mm diameter, with a target thickness of 62 ± 1 mm for CTI testing.

### 2.3. Ideal-CT Test Procedure

**Figure 2:**
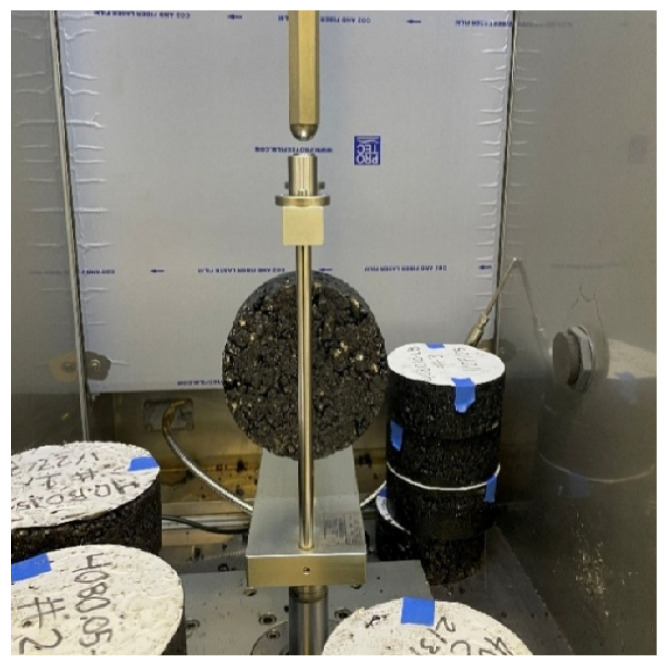
Ideal-CT specimen for testing.

The IDEAL-CT test was executed following the ASTM D8225-19 standard [17], “Standard Test Method for Determination of Cracking Tolerance Index of Asphalt Mixture Using the Indirect Tensile Cracking Test at Intermediate Temperature”. Three trial specimens were tested for each type of mixture. The procedure began with conditioning the specimens in an environmental chamber for a period of 120 ± 10 min prior to testing. The intermediate test temperature (PGIT) was calculated using Equation (1):

(1) $$PG,IT=\frac{PG,HT+PG,LT}{2}+4$$

where PGHT is the climatic high-performance grade temperature (°C) and PGLT is the climatic low-performance grade temperature (°C). This calculation resulted in a test temperature of 31 °C for the 15% RAP mixes (using PG 64-10 binder) and 22 °C for the 25% and 40% RAP mixes (using PG 58-22 binder). A Pavetest Dynamic Loading System was employed for the tests. Each specimen was centrally positioned within the indirect tensile testing fixture as shown in Figure 2. This fixture featured concave steel loading strips with a radius of curvature closely matching that of the specimen to ensure uniform load distribution. Loading plates and fixtures were meticulously cleaned before each test. An axial load was applied to the specimen at a constant Load-Line Displacement (LLD) rate of 50 mm/min.

Because IDEAL-CT was run at PGIT for each binder grade (31 °C for PG 64-10; 22 °C for PG 58-22), CTIndex values are most appropriately compared within a mixture/binder group rather than across mixtures with different PG and test temperatures. Loading continued until the specimen experienced complete failure, defined as the point when the measured load dropped below 100 N. The entire testing process for each specimen, from removal from the conditioning chamber to completion of the test, was maintained within 4 min to minimize temperature variations. Load and displacement data were captured continuously using a high-frequency data acquisition system, recording at a minimum sampling rate of 40 data points per second to generate precise load–displacement curves.

### 2.4. Calculation Parameters

From the acquired load–displacement data, several parameters were computed. The Work of Failure (Wf), which represents the energy (in Joules) required to induce cracking, was calculated as the area under the load–displacement curve using Equation (2):

(2) $$W_{f}=\sum_{i=1}^{n-1}\left[\left(l_{i+1}-l_{i}\right)P_{i}+½\left(l_{i+1}-l_{i}\right)\left(P_{i+1}-P_{i}\right)\right]$$

where Pi is the applied load (kN) at load step i, and li is the LLD (mm) at step i.

The Failure Energy (Gf), which is the energy (in Joules/m^2^) required to initiate and propagate cracks within the specimen, was calculated using Equation (3):

(3) $$G_{f}=\frac{W_{f}}{D*t}\times 10^{6}$$

where D is the specimen diameter (mm) and t is the specimen thickness (mm).

The Cracking Tolerance Index (CTIndex), the major parameter representing the asphalt mixture’s resistance to fatigue cracking, was calculated using Equation (4):

(4) $$CTIndex=\frac{t}{62}\times \frac{l_{75}}{D}\times \frac{G_{f}}{\left|m_{75}\right|}\times 10^{6}$$

where l75 is the displacement (mm) at 75% of the peak load on the post-peak portion of the curve, and ∣m75∣ is the absolute value of the post-peak slope (N/m) of the straight line connecting 85% and 65% of the peak load. A higher CTIndex value signifies better cracking resistance.

## 3. Results

Detailed numerical data and analysis for the different mixtures used in the study are presented in the subsequent sections.

### 3.1. Mixtures with 15% RAP

**Figure 3:**
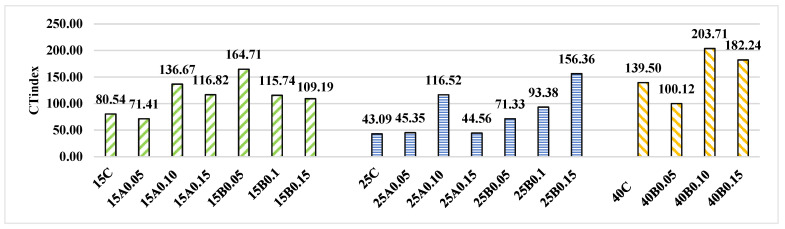
Ideal-CT results for all the mixes (Rahim et al. 2025 [6]).

The cracking resistance of the 15% RAP mixtures varied with the addition of fibers, as shown by the CTIndex values in Figure 3. The control mixture without any fiber established a baseline performance with a CTIndex of 80.5. The addition of Fiber A at a 0.05% dosage was not found to improve CTIndex values, resulting in a lower CTIndex of 71.4. In contrast, all other fiber-modified mixtures outperformed the control. The most substantial improvement was observed in the mixture containing 0.05% of Fiber B, which achieved a CTIndex of 164.7, more than double the value of the control mix. Fiber A at 0.10% and 0.15% yielded CTIndex values of 136.7 and 116.8, representing increases of 70% and 45% over the control (80.5), respectively. Fiber B at 0.10% and 0.15% produced CTIndex values of 115.7 and 109.2, gains of 44% and 36% over the control. Comparing fiber types at equivalent dosages, Fiber B strongly outperformed Fiber A at 0.05% (164.7 vs. 71.4), while Fiber A outperformed Fiber B at 0.10% (136.7 vs. 115.7) and 0.15% (116.8 vs. 109.2), suggesting that the wax-coated fiber becomes more effective at higher dosages where sufficient thermal energy is available to melt the coating and improve fiber–binder bonding.

**Figure 4:**
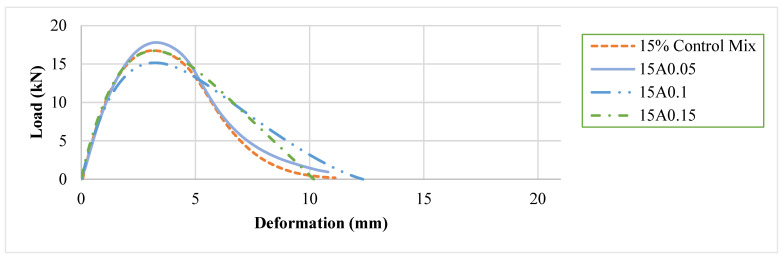
Load–displacement curves for 15% RAP mixes with Fiber A (Rahim et al. 2025 [6]).

The load–deformation curves provided a graphical representation of these performance differences. As depicted in Figure 4, the control mix exhibited the sharpest post-peak drop in load, while all mixes modified with Fiber A showed more extended deformation. The mix with 0.10% Fiber A demonstrated both a higher peak load and a more gradual decline, indicating increased energy absorption. The 0.15% Fiber A mix maintained a similar peak load but had a steeper post-peak slope than the 0.10% dosage.

**Figure 5:**
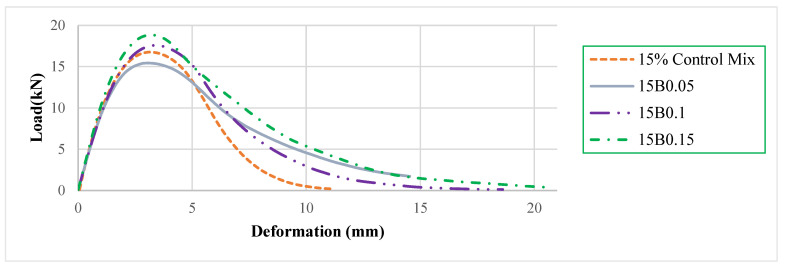
Load–displacement curves for 15% RAP mixes with Fiber B (Rahim et al. 2025 [6]).

The performance of Fiber B mixes relative to the control is shown in Figure 5. All three Fiber B mixes displayed broader deformation ranges than the control mix. The 0.05% dosage exhibited a high peak and the most extended post-peak curve, while the 0.10% and 0.15% dosages reached even higher peak loads but with steeper post-peak slopes. The 0.15% mix achieved the highest peak compared to the other mixtures in this group.

### 3.2. Mixtures with 25% RAP

The results for the 25% RAP mixtures are presented in Figure 3. The control mix for this group had a relatively low CTIndex of 43.09, which established the baseline for cracking resistance. Fiber A at 0.05% and 0.15% produced CTIndex values of 45.4 and 44.6, representing negligible gains of +5% and +3% over the control (43.1). In contrast, Fiber A at 0.10% produced a CTIndex of 116.5, a 170% increase over the control (*p* = 0.002), highlighting a sharp and narrow optimal dosage window for wax-coated fiber in this mix. For the mixtures modified with Fiber B, there was a clear trend of increasing CTIndex with higher dosages. Fiber B at 0.05% and 0.10% produced CTIndex values of 71.3 (+66%) and 93.4 (+117%) over the control, respectively. Fiber B at 0.15% achieved 156.4, a 263% increase over the control (*p* = 0.018) and the highest CTIndex in the 25% RAP group, 34% higher than the best Fiber A result (116.5 at 0.10%). Across both fiber types, the 25% RAP group showed the largest absolute and relative improvements from fiber addition, consistent with its low baseline cracking resistance.

**Figure 6:**
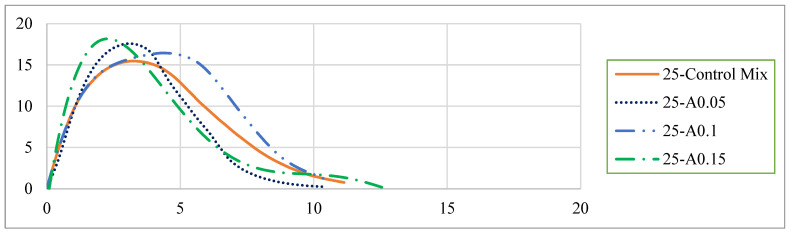
Load–displacement curves for 25% RAP mixes with Fiber A (Rahim et al. 2025 [6]).

**Figure 7:**
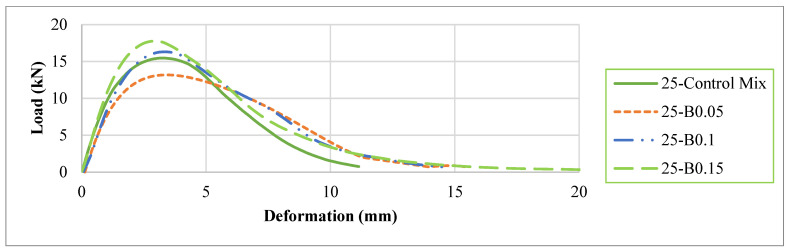
Load–displacement curves for 25% RAP mixes with Fiber B (Rahim et al. 2025 [6]).

The load versus deformation curves in Figure 6 illustrate the performance of the Fiber A mixtures. The 0.10% Fiber A mix demonstrated the most favorable performance, maintaining a relatively high load over a broader deformation range, which indicates improved ductility. In contrast, the 0.05% and 0.15% Fiber A mixes reached higher peak loads than the control but exhibited sharper post-peak declines, suggesting a more brittle response. The curves in Figure 7 show the behavior of the Fiber B mixtures. Among these, the 0.15% Fiber B mix exhibited the most favorable response, achieving the highest peak load and maintaining strength over the widest deformation range, indicating superior cracking resistance and toughness. The 0.05% Fiber B mix showed a lower peak load and a sharp decline after reaching its maximum, suggesting a more brittle failure mode.

### 3.3. Mixtures with 40% RAP

**Figure 8:**
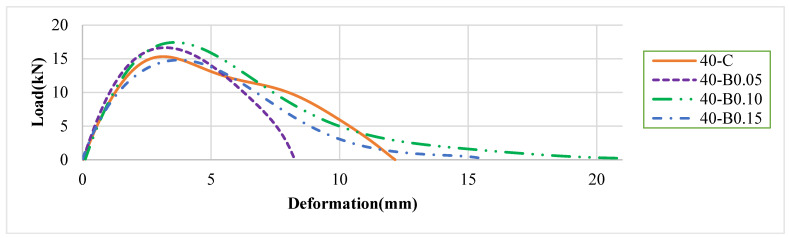
Load–displacement curves for 40% RAP mixes with Fiber B (Rahim et al. 2025 [6]).

Fiber B showed better performance at 15% and 25% RAP, so only Fiber B was tested at 40% RAP to focus on the more effective modifier. As the RAP content increased to 40%, the baseline cracking resistance of the control mix was surprisingly high, with a CTIndex of 139.50 (see Figure 3). The addition of Fiber B (the only fiber tested at this RAP level) produced mixed results. A low dosage of 0.05% was counterproductive, reducing CTIndex from 139.5 to 100.1 (−28%), suggesting that insufficient fiber content in a stiff, high-RAP matrix may disrupt aggregate contact without providing meaningful crack-bridging benefit. Higher dosages substantially enhanced performance: the 0.10% Fiber B mix achieved a CTIndex of 203.7 (+46% vs. control), the highest mean value of any mix tested across all three RAP levels. The 0.15% Fiber B mix reached 182.2 (+31% vs. control). None of these changes were statistically significant (*p* = 0.762, 0.911, and 0.928 for 0.10%, 0.15%, and 0.05% respectively), primarily due to high specimen variability with coefficients of variation ranging from 36% to 64%. The load–displacement curves for the 40% RAP control and Fiber B mixtures are shown in Figure 8.

## 4. Discussion and Analysis

**Figure 9:**
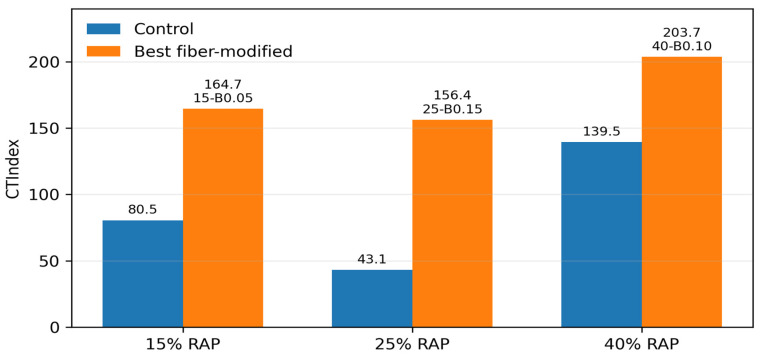
Comparative CTIndex summary for control vs. best-performing fiber mix within RAP group.

Mechanistically, RAP increases stiffness through aged binder, while fibers improve cracking resistance by bridging cracks after initiation. Effective dosages increase fracture energy and reduce post-peak slope, but too little or poorly dispersed fiber may limit crack-bridging benefits. A comprehensive assessment revealed that the relationship between fiber dosage and cracking performance is not linear and is highly dependent on both the fiber type and the RAP content of the mixture. As evident in Figure 9 and Table 2, in the 15% RAP mixes, the superior performance of Fiber B at a low 0.05% dosage, which more than doubled the CTIndex from 80.5 to 164.7, is particularly noteworthy. This could be attributed to the liquid emulsion treatment on Fiber B, which may promote more uniform dispersion and a stronger fiber–binder bond at lower concentrations.

The CTIndex improvement can also be interpreted through its calculation parameters. A higher CTIndex may result from increased fracture energy (Gf), larger post-peak displacement (l75), a lower absolute post-peak slope (|m75|), or a combination of these factors. As shown in Table 2, the selected fiber-modified mixtures generally had higher fracture energy and lower post-peak slope values than their corresponding control mixtures. This indicates that the improvement was mainly related to greater energy absorption and a more gradual post-peak load reduction. Although l75 did not increase for every selected mixture, the combined effects of Gf, l75, and |m75| produced higher CTIndex values. Fibers increase CTIndex by making the mixture less brittle after peak load. They bridge developing cracks, allowing the specimen to carry load over a larger deformation range. This increases fracture energy and post-peak deformation while reducing the steepness of the post-peak slope. As a result, the load–deformation curve becomes broader and flatter after peak load, indicating better cracking resistance.

In contrast, the wax-coated Fiber A performed worse at this same low dosage, suggesting its coating may require a higher dosage and associated thermal energy to effectively melt and facilitate a proper bond within the matrix. The performance peak for Fiber A at 0.10% in both the 15% and 25% RAP mixes further supports the concept of an optimal dosage window, beyond which the benefits may plateau or, as seen with the 40% RAP mix, even reverse due to potential fiber cluster or stiffening effects.

The load–displacement curves in Figure 4, Figure 5, Figure 6, Figure 7 and Figure 8 were compared to better explain the CTIndex results. Overall, the control mixtures showed sharper post-peak load drops, indicating more brittle cracking after crack initiation. In contrast, the fiber-modified mixtures with higher CTIndex values generally maintained load over a wider displacement range, showing improved post-peak toughness and energy absorption. This behavior was most evident for 15-B0.05, 15-A0.10, 25-A0.10, 25-B0.15, and 40-B0.10. These mixtures suggest that fibers improved cracking resistance mainly through crack-bridging and delayed crack propagation, rather than only by increasing peak load. However, the 40% RAP results must be interpreted cautiously as high variability reduced statistical confidence.

Table 3 shows statistical analysis results using a one-way ANOVA and Tukey’s Honest Significant Difference (HSD) test, performed to evaluate the significance of the fibers’ effect on performance when compared to the control group and the coefficient of variation within the sample set of each mix. For the 15% RAP mixes, a statistically significant improvement was found only at a 0.10% dosage for Fiber A (*p* = 0.0307) and at a 0.05% dosage for Fiber B (*p* = 0.0255). In 25% RAP mixes, Fiber A at 0.10% increased CTIndex to 116.5 (*p* = 0.002). The 0.05% and 0.15% Fiber A mixes were not significantly different from control (*p* ≈ 1.00). Fiber B showed a monotonic mean increase with dosage, but only 0.15% reached statistical significance versus control (156.4; *p* = 0.0177). For the 40% RAP mixes, although the 0.10% and 0.15% Fiber B dosages increased the mean CTIndex), none of the fiber dosages were statistically different from the control (*p* > 0.05), likely due to higher variability (CV36–64%).

## 5. Limitations

This study was limited to laboratory evaluation using the IDEAL-CT test. Although IDEAL-CT provides useful information about intermediate-temperature cracking resistance, it is an index-based test and does not fully represent field cracking performance. Additional mechanistic tests, such as fatigue testing, semi-circular bending, overlay testing, Hamburg wheel tracking, moisture susceptibility testing, and long-term aging evaluation, are needed to confirm the broader performance of the fiber-modified RAP mixtures. Therefore, the conclusions should be interpreted as IDEAL-CT screening results rather than complete balanced mix design recommendations.

Only three replicate specimens were tested for each condition, which may have contributed to statistical uncertainty, especially in the 40% RAP mixtures where the coefficient of variation was high. No quantitative workability test, such as mixing torque, compaction effort index, coating evaluation, or plant-production workability assessment, was performed. In addition, the mixtures were obtained from different sources and used different binder grades and test temperatures. Therefore, comparisons should be interpreted primarily within each RAP group rather than as direct comparisons across all RAP levels. Field validation is also needed before recommending broad implementation of the fiber-modified mixtures.

## 6. Conclusions

This study evaluated the effect of two commercial para-aramid fiber products on the IDEAL-CT cracking resistance of HMA mixtures containing 15%, 25%, and 40% RAP. The results showed that fiber reinforcement can improve laboratory cracking resistance, but the improvement depends strongly on RAP content, fiber treatment, and dosage.

- For the 15% RAP mixture, both fiber products improved CTIndex when used at an effective dosage. Fiber B at 0.05% and Fiber A at 0.10% produced statistically significant improvements compared with the control. This indicates that lower RAP mixtures may benefit from fiber reinforcement, but the optimum dosage depends on fiber surface treatment and dispersion behavior.
- For the 25% RAP mixture, fiber modification produced the greatest relative improvement. The control mixture had the lowest CTIndex, and the addition of fibers substantially improved cracking tolerance. Fiber A performed best at 0.10%, while Fiber B showed its best performance at 0.15%. This suggests that mixtures with lower baseline cracking resistance may benefit most from properly dosed fiber reinforcement.
- For the 40% RAP mixture, Fiber B at 0.10% produced the highest mean CTIndex among all mixtures tested. However, the improvement was not statistically significant because of high variability among replicate specimens. Therefore, the 40% RAP results should be interpreted cautiously and should be verified using additional replicates and complementary performance tests.

The findings demonstrate that fiber dosage should be optimized for each RAP mixture rather than selected as a fixed universal dosage. The results also suggest that fiber reinforcement primarily improves cracking performance by enhancing post-peak toughness, increasing deformation tolerance, and delaying crack propagation. Future work should include workability evaluation, fatigue performance, moisture susceptibility, long-term aging, fiber dispersion evaluation, economic analysis, and field validation. Because only IDEAL-CT was used, the results should be used to identify candidate dosages for further evaluation rather than as final mixture design recommendations.

## Figures and Tables

**Table 1:** Gradation of combined aggregate blend for the mixtures used in the study.

Sieve Size	15% RAP (% Passing)	25% RAP (% Passing)	40% RAP (% Passing)
1 in	100	100	100
¾ in	100	98	98
½ in	97	83	82
⅜ in	83	74	73
#4	51	52	51
#8	38	33	32
#16	27	22	22
#30	18	15	16
#50	11	10	11
#100	6	6	8
#200	3.3	4.3	5.5

**Table 2:** Key CTIndex variables for control and best-performing mixes.

Mix	Gf (J/m^2^)	l75 (mm)	|m75| × 10^6^ (N/m)	CTIndex
15-C	9622	5.22	4.38	80.54
15-B0.05	11,841	5.87	2.86	164.71
25-C	10,055	5.19	8.5	43.09
25-B0.15	14,379	6.05	3.71	156.36
40-C	11,948	6.39	3.66	139.5
40-B0.10	14,004	5.86	2.96	203.71

**Table 3:** Statistical significance of the CTI_ndex_ values of the modified mixes compared to control mix.

RAP %	Fiber Type	0.05% Fiber		0.10% Fiber		0.15% Fiber	
		*p*-Value (*p* ≤ 0.05 Significant?)	Coefficient of Variance (%)	*p*-Value (*p* ≤ 0.05 Significant?)	Coefficient of Variance (%)	*p*-Value (*p* ≤ 0.05 Significant?)	Coefficient of Variance (%)
15%	A	0.936 (No)	18	0.031 (Yes)	19	0.178 (No)	10
15%	B	0.026 (Yes)	17	0.459 (No)	19	0.613 (No)	34
25%	A	0.998 (No)	61	0.002 (Yes)	31	0.999 (No)	11
25%	B	0.810 (No)	23	0.432 (No)	53	0.018 (Yes)	33
40%	B	0.928 (No)	36	0.762 (No)	50	0.911 (No)	64

## Data Availability

The original contributions presented in this study are included in the article. Further inquiries can be directed to the corresponding author.
